# CLIBASIA_00460 Disrupts Hypersensitive Response and Interacts with Citrus Rad23 Proteins

**DOI:** 10.3390/ijms23147846

**Published:** 2022-07-16

**Authors:** Junepyo Oh, Julien G. Levy, Chia-Cheng Kan, Freddy Ibanez-Carrasco, Cecilia Tamborindeguy

**Affiliations:** 1 Department of Entomology, Texas A&M University, College Station, TX 77843, USA; ohjunepyo@tamu.edu (J.O.); cc.kan@okstate.edu (C.-C.K.); 2 Department of Horticultural Sciences, Texas A&M University, College Station, TX 77843, USA; 3 Department of Entomology, Texas A&M AgriLife Research, Weslaco, TX 78596, USA; freddy.ibanez-carras@ag.tamu.edu

**Keywords:** psyllid, *Candidatus* Liberibacter solanacearum, Huanglongbing, citrus greening, effector

## Abstract

‘*Candidatus* Liberibacter asiaticus’ (CLas) is a bacterium that causes Huanglongbing, also known as citrus greening, in citrus plants. ‘*Candidatus* Liberibacter solanacearum’ (Lso) is a close relative of CLas and in the US it infects solanaceous crops, causing zebra chip disease in potato. Previously, we have identified the Lso hypothetical protein effector 1 (Lso-HPE1). This protein uses a signal peptide for secretion; disrupts programmed cell death; and interacts with tomato RAD23c, d, and e proteins, but not with RAD23a. In this study, we evaluated whether CLIBASIA_00460, the CLas homolog of Lso-HPE1 interacted with citrus RAD23 proteins and disrupted their programmed cell death. Based on the yeast two-hybrid assay results, CLIBASIA_00460 interacted with citrus RAD23c and RAD23d, but not with citrus RAD23b. These results were confirmed using bimolecular fluorescence complementation assays, which showed that these interactions occurred in cell puncta, but not in the nucleus or cytoplasm. Additionally, CLIBASIA_00460 was able to disrupt the Prf^D1416V^-induced hypersensitive response. Therefore, based on the similar interactions between Lso-HPE1 and CLIBASIA_00460 with the host RAD23 proteins and their ability to inhibit cell death in plants, we propose that these effectors may have similar functions during plant infection.

## 1. Introduction

‘*Candidatus* Liberibacter asiaticus’ (CLas) is the causative agent of citrus greening or Huanglongbing (HLB), the most devasting disease of citrus fruits [1,2]. This disease arrived in Florida in the early 2000s and has spread throughout the citrus-producing areas of Florida, California, and Texas. Since 2005, HLB has reduced citrus production by 75%, taking an unprecedented toll on major fruit production [3]. Typically, during the early years following the arrival of the disease, infected trees were destroyed. Currently, there is no treatment for trees infected with HLB.

CLas is a Gram-negative α-proteobacterium [4,5], and is the causative agent of HLB in the USA. Two other bacteria, ‘*Ca.* L. africanus’ and ‘*Ca*. L. americanus’ [6,7] also cause HLB throughout the world, but CLas is the most prevalent and virulent of these pathogens [4]. CLas colonizes the phloem sieve elements of citrus plants. In nature, CLas is transmitted to plants through feeding by the Asian citrus psyllid, a phloem-feeding insect. HLB symptoms include leaf mottling, discolored fruit, and aborted seeds, eventually leading to the death of the entire tree [8].

In the last 20 years, other *Liberibacter* species have emerged as pathogens affecting many crops in different parts of the world. For example, ‘*Candidatus* Liberibacter solanacearum’ (Lso) haplotypes A and B affect potato, tomato, and other solanaceous crops in the Americas, causing zebra chip disease in potato [9]. In New Zealand, only haplotype A is present. Lso haplotypes C, D, and E affect carrot and celery in Europe, North Africa, and in the Middle East [10,11,12,13]. Other Lso haplotypes have also been identified elsewhere, infecting other plant species [14,15]. More recently, Lso was found to be associated with cottony ash psyllids and could be responsible for the ash tree decline in Canada [16]. Similar to other *Liberibacter* pathogens, Lso is a phloem-restricted bacterium that is transmitted by psyllids. Given that the diseases that are caused by Lso develop faster than those which are not, we propose to use Lso as a model pathosystem to investigate the diseases caused by *Liberibacter* pathogens.

The complete genomes of several *Liberibacter* species have been sequenced through metagenomic [17,18,19,20,21] and transcriptome analyses [22,23], paving the way for functional genomics studies. Specifically, several research groups have investigated the biological functions of the *Liberibacter*-encoded proteins in relation to their role in plants [24]. *Liberibacter* are intracellular pathogens that lack the type III, IV, or VI secretion systems (T3SS, T4SS, and T6SS, respectively), which are well known for injecting effector proteins into the host’s cells. As a result, the general Sec secretion machinery is believed to be responsible for the secretion of the *Liberibacter* effectors [25]. When using *Escherichia coli* as a surrogate system, several *Liberibacter* proteins have been shown to potentially be secreted by the Sec system [26]. Most of the Sec-dependent secreted proteins that are encoded by *Liberibacter* bacteria are annotated as hypothetical proteins.

In a previous work, we identified Lso-HPE1 (ADR52633.1), an effector that is encoded by Lso. Lso-HPE1 is a Sec-dependent secreted protein that is able to suppress a hypersensitive response (HR) when induced by either BAX or Prf^D1416V^ in *Nicotiana benthamiana* [27]. The screening of a tomato yeast two-hybrid library identified tomato RAD23e as an interactor of Lso-HPE1 [28]. RAD23 proteins are involved in shuttling ubiquitinated proteins to the proteasome for them to undergo degradation. Tomato encodes four RAD23 genes (RAD23a, RAD23c, RAD23d, and RAD23e) and Lso-HPE1 can interact with tomato RAD23c, RAD23d, and RAD23e, but not with RAD23a. While Lso-HPE1 is well conserved among the different Lso haplotypes, the CLas homolog protein identified, CLIBASIA_00460, shares 34% identity with Lso-HPE1 and has 55% similarity with it. These values increase to 46% and 60% for identity and similarity when compared to the mature protein, i.e., when the signal peptide is removed [27]. No homologs of Lso-HPE1 were found in ‘*Ca.* L. africanus’ or ‘*Ca.* L. americanus’.

The purpose of this study was to evaluate whether CLIBASIA_00460, the CLas protein homolog of Lso-HPE1, could also suppress HR induced by Prf^D1416V^ in *N. benthamiana*, and if it could interact with citrus RAD23 proteins.

## 2. Results

A 98-amino-acid-long putative Lso-HPE1 homolog was identified in the CLas genome, CLIBASIA_00460 (WP_012778427) [27]. BLAST searches using CLIBASIA_00460 as a query returned as match CLas hypothetical proteins (WP_012778427) and Lso-HPE1 proteins from different Lso haplotypes. However, no similar proteins were identified in ‘*Ca*. L. africanus’, ‘*Ca*. L. americanus’, ‘*Ca*. L. europaeus’, or *L. crescens*. CLIBASIA_00460 has a predicted signal peptide [26,29,30]. CLIBASIA_00460 protein sequences are identical in the 62 CLas assembly accessions available in NCBI. These accessions correspond to CLas specimens collected from different geographical regions.

**Figure 1:**
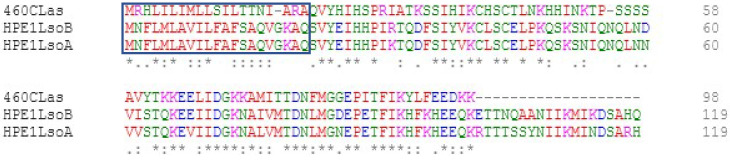
Alignment of the LsoA and LsoB HPE1 and CLIBASIA_00460 proteins. The signal peptide is boxed. The amino acid residues are colored according to their physiochemical properties: red = small (small + hydrophobic including aromatic − Y), blue = acidic, magenta = basic − H, and green = hydroxyl + sulfhydryl + amine + G. The consensus symbols are as follows: an asterisk (*) indicates positions which have a single, fully conserved residue; a colon (:) indicates conservation between groups with strongly similar properties; and a period (.) indicates conservation between groups with weakly similar properties.

While CLas CLIBASIA_00460 is shorter than Lso-HPE1 proteins as it lacks the last 21 amino acids, the C-terminal region of the protein is highly conserved (Figure 1). Both mature proteins have a predicted subcellular localization in the nucleus and the cytoplasm (Table S1), which is in accordance with the localization observed in expression assays [29].

### 2.1. Suppression of Plant Cell Death

**Figure 2:**
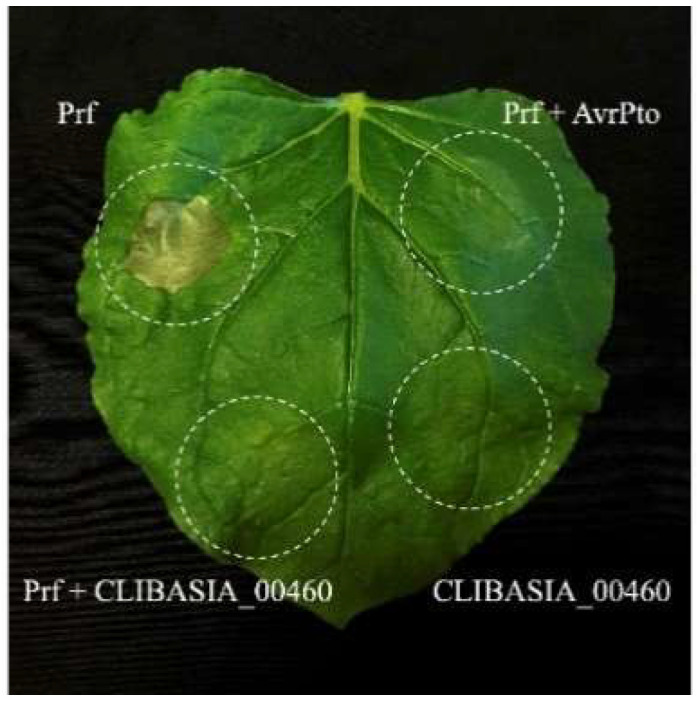
Symptoms associated with transient expression in *Nicotiana benthamiana* leaves after agroinfiltration. The picture was taken seven days after infiltration. Prf^D1416V^ alone (Prf) induced cell death, while the co-infiltration of Prf^D1416V^-AvrPto, the co-infiltration of Prf^D1416V^-CLIBASIA_00460, or the infiltration of CLIBASIA_00460 alone did not induce cell death.

The transient expression of the mature CLIBASIA_00460 in *N. benthamiana* did not induce cell death (average score of 0.03). However, the CLIBASIA_00460 mature protein was able to suppress Prf^D1416V^-induced cell death in co-infiltration assays (average score of 0.18). The average score for Prf^D1416V^ when it was expressed alone was 0.55 (cell death control), and when it was co-infiltrated with AvrPto, the score was 0 (suppression of cell death control) (Figure 2).

### 2.2. Identification of RAD23 Proteins Encoded in the Citrus Genome

**Figure 3:**
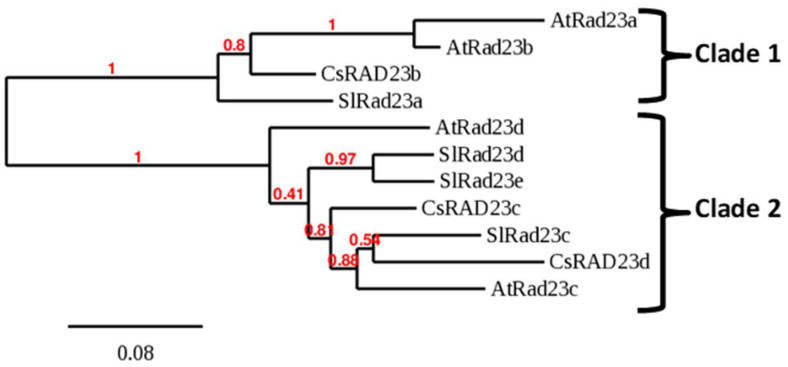
Phylogenetic tree of the RAD23 proteins. The tree was obtained using the “one click” mode in phylogeny.fr.

**Figure 4:**
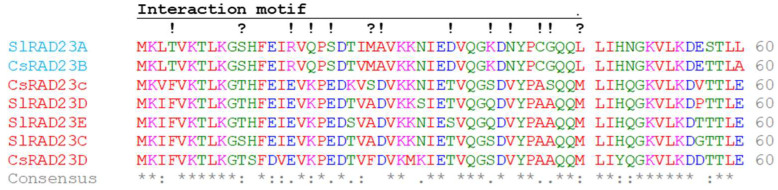
Alignment of the *S. lycopersicum* and *C. sinensis* RAD23 proteins. The names of the sequences in Clade 1 are in blue; those in Clade 2 are in red. Amino acid residues are colored according to their physiochemical properties: red = small (small + hydrophobic including aromatic − Y), blue = acidic, magenta = basic − H, and green = hydroxyl + sulfhydryl + amine + G. Consensus symbols are as follows: an asterisk (*) indicates the positions which have a single, fully conserved residue; a colon (:) indicates conservation between groups of strongly similar properties; and a period (.) indicates conservation between groups of weakly similar properties. The motif interacting with Lso-HPE1 is identified using a line; the symbols below the line are as follows: a question mark (?) indicates the positions with amino acid substitutions with similar physiochemical properties between clades, and an exclamation mark (!) indicate the positions with amino acid substitutions with different physiochemical properties between clades.

Citrus RAD23 proteins were identified using BLAST searches employing tomato and *Arabidopsis thaliana* RAD23 as the query. We searched two citrus genomes, *Citrus clementina* and *C. sinensis,* to find similar proteins. In each species, three RAD23 genes were identified (RAD23b, RAD23c, and RAD23d). The phylogenetic tree constructed using the *Solanum lycopersicum*, *A. thaliana,* and *C. sinensis* proteins shows two RAD23 clades (Figure 3). Clade 1 clusters *A. thaliana* RAD23a and b; *C. sinensis* RAD23b; and *S. lycopersicum* RAD23a proteins. The second clade, Clade 2, clusters *A. thaliana* RAD23c and d; *S. lycopersicum* RAD23c, d, and e; and *C. sinensis* RAD23c and d proteins. A multiple sequence alignment of the RAD23 domain interacting with Lso-HPE1 is shown in Figure 4; the complete alignment of the RAD23 proteins is shown in Figure S1 (a phylogenetic analysis of this family can be found in [31]). Based on the bioinformatic analyses, all *S. lycopersicum* and *C. sinensis* RAD23 proteins were predicted to be localized in the nucleus and cytoplasm (Table S1).

### 2.3. Yeast Two-Hybrid Interaction between CLIBASIA_00460 and Citrus RAD23

**Figure 5:**
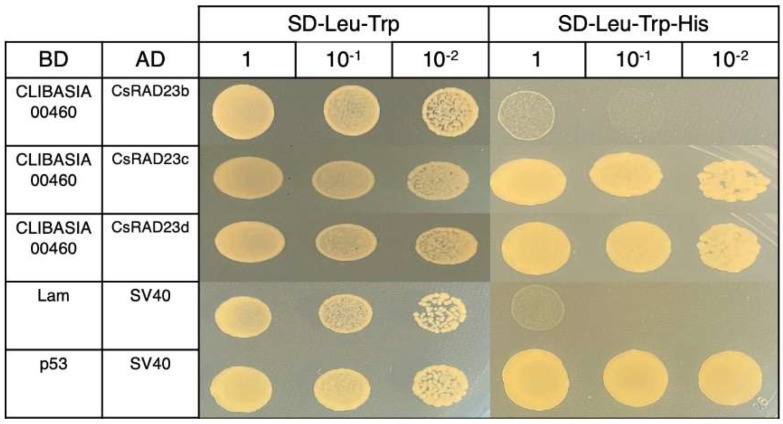
Evaluation of the interaction between the CLIBASIA_00460 mature protein and the citrus RAD23 proteins using directed yeast two-hybrid assays. pGBKT7 (BD) constructs were used as bait, and pGADT7 (AD) constructs were used as prey in the yeast two-hybrid assays. Lam + SV40 indicates the negative control for the yeast-two hybrid assays; p53 + SV40 indicates a positive control for the yeast two-hybrid assays.

To evaluate whether CLIBASIA_00460 interacted with citrus RAD23 proteins, yeast two-hybrid assays were performed. The CLIBASIA_00460 mature protein interacted with the *C. sinensis* RAD23c and RAD23d proteins but did not interact with the *C. sinensis* RAD23b protein (Figure 5).

### 2.4. Validation of Interaction by Bi-Molecule Fluorescence Complementation

**Figure 6:**
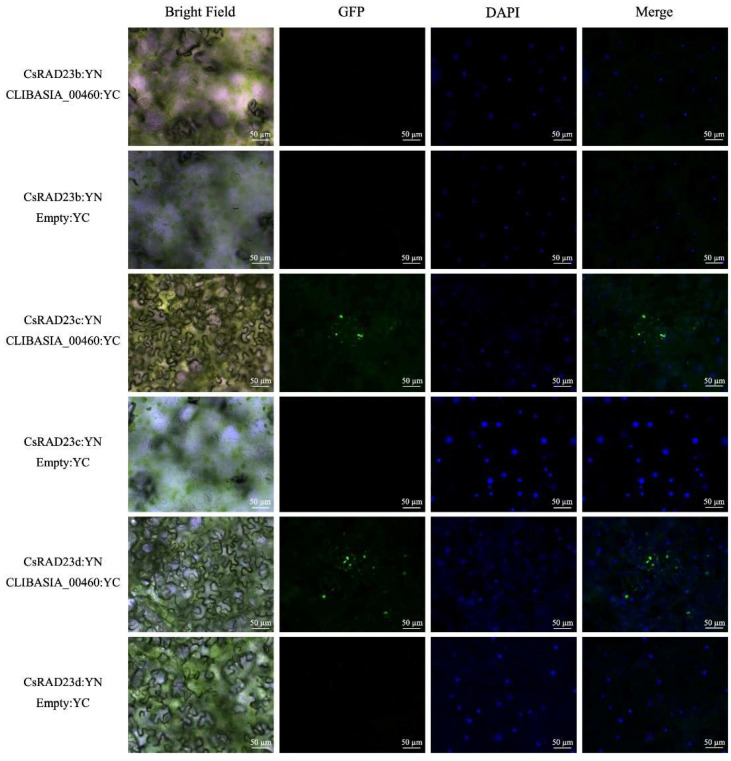
Bi-molecule fluorescence complementation analysis of mature CLIBASIA00460 and citrus RAD23b, c, and d interaction in *N. benthamiana* epidermal cells that are infiltrated with a 1:1 ratio of agrobacteria containing YC:CLIBASIA_00460 and YN:RAD23b, c, or d. Also shown are the negative controls which were obtained using infiltration with a 1:1 ratio of agrobacteria containing YC:empty and YN:RAD23b, c, or d. The images were taken between 72 and 80 h post-infiltration. Scale bar = 50 µm.

The interaction between CLIBASIA_00460 and the *C. sinensis* RAD23 proteins was validated using Bi-molecule fluorescence complementation (BiFC). To validate the interaction between the CLIBASIA_00460 mature protein and *C. sinensis* RAD23, YFP fluorescence signals in infiltrated leaf cells were visualized using fluorescence microscopy. In accordance with the results obtained in the directed Y2H assays, fluorescence signals were observed in the leaf cells that were co-infiltrated with the proteins CsRAD23c:YN and CLIBASIA_00460:YC, or CsRAD23d:YN and CLIBSIA_00460:YC, but no signal was observed in those where CsRAD23b:YN and CLIBASIA_00460:YC were co-infiltrated (Figure 6). The interaction between CsRAD23c:YN or CsRAD23d:YN and CLIBASIA_00460:YC produced discrete signals, but no signal was observed in the nucleus and cytoplasm. The mature CLIBASIA_00460, upon transient expression, was localized at the nucleus and cytosol of the epidermal cells (Figure S2).

## 3. Discussion

RAD23 proteins are involved in the turnover of plant proteins, which is an essential component of a plant’s defenses [32]. At present, these proteins are emerging as key targets of bacterial effectors. For example, the phytoplasma effector SAP54 targets the host plant’s RAD23 proteins, leading to the degradation of the MADS-domain transcription factors and the development of typical symptoms associated with phytoplasma infection [33]. In our previous study, we identified the effector Lso-HPE1, which can modulate a plant’s defense mechanisms and interact with *S. lycopersicum* RAD23 proteins. Further, we showed that in Lso-infected plants that overexpress Lso-HPE1, Lso detection could be performed earlier and that symptoms of Lso also develop earlier compared to in wild-type infected tomato plants. Therefore, we hypothesized that CLas could encode a protein effector similar to Lso-HPE1 that could interact with citrus RAD23 proteins.

The search for CLas homologs of Lso-HPE1 identified CLIBASIA_00460, which was predicted to be secreted via the Sec system. The search for homologs of Lso-HPE1 did not identify similar proteins in the other *Liberibacter* pathogens that cause HLB or in the culturable *L. crescens*. A reciprocal search using CLIBASIA_00460 as a query confirmed these results. Thus, it appears that while CLIBASIA_00460 might not be essential for citrus infection, it could be linked to the higher aggressivity of CLas. Alternatively, the other *Liberibacter* pathogens pathogens could rely on a different effector protein with a similar function to that of CLIBASIA_00460.

While the HPE1 protein encoded by different Lso haplotypes is highly conserved, it is not identical. The differences in the sequences could be associated with the difference in host range among the haplotypes [27], or a difference in virulence [34,35]. However, all CLas CLIBASIA_00460 proteins are identical in the 62 assembly accessions available from NCBI. These accessions were obtained by sequencing CLas-infected samples from different geographical regions.

Our search for citrus RAD23 proteins identified three genes in *C. sinensis* and *C. clementina*. The phylogenetic analysis determined that the *C. sinensis* RAD23b protein clustered with *A. thaliana* RAD23a and b (Clade 1 in Figure 2). This clade also contained *S. lycopersicum* RAD23a. The other two *C. sinensis* RAD23 proteins, RAD23c and d clustered with *A. thaliana* RAD23c and d, as well as *S. lycopersicum* RAD23c, d, and e (Clade 2). Previous phylogenetic analyses of RAD23 proteins identified two separate clades in higher plants which arose after the evolution of seed plants but before the monocot/eudicot split [31].

The CLIBASIA_00460 mature protein interacted with *C. sinensis* RAD23c and d in a yeast two-hybrid assay, but not with *C. sinensis* RAD23b. These results were confirmed using BiFC. An analysis of Lso HPE1 and *S. lycopersicum* RAD23 proteins also identified the interaction between the effector protein and the RAD23 proteins in Clade 2, but not in Clade 1 [28]. A mutant analysis determined that in *A. thaliana*, at least one functional RAD23 gene is required for plant viability, independent of the RAD23 subfamily [31]. While these proteins might have overlapping functions, it was determined that each RAD23 protein had some specific roles—for example, RAD23a during embryogenesis; RAD23b in leaf phyllotaxy, root growth, apical dominance, and fertility; and RAD23c in fertilization [31]. Based on our results, the RAD23 proteins in Clade 2 might be targeted by Liberibacter effectors, and this interaction could be involved in pathogenicity. Interestingly, the phytoplasma effector SAP54 also interacts with the *A. thaliana* RAD23 proteins in Clade 2, but not in Clade 1. In light of these results, we hypothesize that RAD23 Clade 2 might play a role in plant defenses.

The CLIBASIA_00460 mature protein had a different localization in transient expression experiments, as reported here (Figure S2) and in other studies [29,36], than when its interaction with *C. sinensis* RAD23 proteins was assessed using BiFC. Indeed, the mature effector showed a nuclear and cytoplasmic localization in transient expression experiments, while fluorescent signals were only observed in the puncta in the BiFC experiments. Thus, the subcellular localization pattern was changed when CLIBASIA_00460 interacted with *C. sinensis* RAD23c or RAD23d proteins. Lso-HPE1 also interacted with *S. lycopersicum* RAD23 proteins, but there was no change in the localization of the proteins; the interaction occurred in the nucleus and cytoplasm [28]. This difference in the localization of CLIBASIA_00460 when interacting with the *C. sinensis* RAD23 proteins was unexpected because the mature Lso-HPE1 and CLIBASIA_00460 proteins, as well as the different RAD23 proteins, were predicted to be localized in the nucleus and the cytoplasm. Previously, the mature CLIBASIA_00460 was shown to be distributed in the cytosol and to accumulate in small vesicles and in elongated shapes [36]. Meanwhile, another study determined that CLIBASIA_00460 was localized in the nucleus and the cytoplasm based on transient expression analyses, but that the subcellular distribution of the protein was affected by temperature, i.e., when the temperature increased to 32 °C, the nuclear accumulation decreased [29]. Overall, these studies show that the localization of CLIBASIA_00460 can be affected by abiotic conditions, and this should be taken into consideration when evaluating the development of HLB under different conditions. Previously, the effect of temperature on subcellular distribution was not observed for Lso-HPE1 [28]. Lso-HPE1 and CLIBASIA_00460 mature proteins share 60% amino acid sequence similarity; these differences could be linked to the differences in protein localization associated with temperature or the interaction with RAD23 proteins. Indeed, these Liberibacter genes could have evolved host-specific functions, so the role of CLIBASIA_00460 in citrus infection should be investigated.

Because of the differences in the localization of CLIBASIA_00460 when it was interacting with RAD23 proteins, we evaluated whether this effector was able to disrupt a HR induced by Prf^D1416V^. The transient expression assays showed that the CLIBASIA_00460 mature protein was able to efficiently disrupt Prf^D1416V^-induced HR. Furthermore, the experiments showed no evidence that the effector induced cell death. Previously, Liu et al. [29] conducted an immune response study of CLIBASIA_00460 in which they overexpressed the mature protein via a Potato virus X (PVX)-based expression vector in *N. benthamiana* samples. They reported that the PVX- CLIBASIA_00460 mature protein induced no visible symptoms in the infiltrated leaves, but from five to six days after infiltration, crinkling and veinal chlorosis were observed in the systemically infected leaves, which later evolved to chlorosis and tiny necrotic spots [29]. Because PVX gRNAs accumulated at a higher level in the PVX-infected plants than in the PVX-CLIBASIA_00460-infected plants, the authors speculated that the observed symptoms might not be related to the over-multiplication of PVX but instead to a heterologously expressed effector, and suggested that CLIBASIA_00460 could have a pathogenic role in planta.

The Lso-solanaceous and CLas-citrus pathosystems present striking similarities and differences; however, few works have performed transversal analyses on them. Maybe these analyses could help uncover the key proteins involved in the infection of the host plants by these pathogens.

## 4. Materials and Methods

### 4.1. Insect Colonies and Tomato Plants

*Nicotiana benthamiana* plants were grown from seed in pots with Sun Gro Sunshine LP5 mix (Bellevue, WA, USA) and fertilized twice a week with Miracle-Gro Water-Soluble Tomato Plant Food, at the label rate (18-18-21 NPK; Scotts Miracle-Gro Company, Marysville, OH, USA). One-week-old seedlings were transplanted to individual pots and were maintained under the same conditions.

*Diaphorina citri* adults were collected from citrus groves located at the Texas A&M AgriLife Research and Extension Center at Weslaco and transported to the laboratory. The presence of CLas was determined from at least 20 insects following the TaqMan qPCR procedure described in Li et al. [37]. Samples showing high CLas infection (CT < 25) were used for the cloning of CLIBASIA_00460.

### 4.2. Sequences Analysis

All the sequence accession numbers used are provided in the text. Homologous sequences were found using BLAST searches (https://blast.ncbi.nlm.nih.gov/Blast.cgi, access date 6 January 2022). A BLASTP search analysis was performed to identify the most similar proteins of Lso-HPE1 in the CLas genome. Then, the CLas protein was used as a query to search for similar proteins in other *Liberibacter* genomes. The prediction of a signal peptide was performed using SignalP-6.0 [38].

RAD23 homologs were identified by BLASTP searches in citrus using *A. thaliana* and *S. lycopersicum* RAD23 sequences. The phylogenetic analysis was performed using the “one click” mode in phylogeny.fr [39]. Briefly, the sequences were aligned with MUSCLE [40] and the alignment was curated with Gblocks [41]. The tree was built with PhyML [42] and drawn with TreeDyn [43].

Multiple sequence alignments of Lso-HPE1 and CLas CLIBASIA_00460 proteins and of the tomato and citrus N-terminal RAD23 protein sequences to identify conserved amino acids were performed using Clustal Omega in EMBL-EBI [44]. The subcellular localization of RAD23 and of the mature Lso-HPE1 and CLIBASIA_00460 proteins was predicted using DeepLoc 2.0 [45].

### 4.3. Directed Yeast Two-Hybrid (Y2H)

For the directed Y2H assay, the sequence encoding the mature protein (without signal peptide) of CLIBASIA_00460 (accession number: ACT55680) was amplified from CLas-infected Asian citrus psyllid DNA and cloned into the pGBKT7 vector as bait. The full-length *C. sinensis* RAD23b, c, and d coding sequences were amplified from citrus cDNA and cloned, separately, into the pGADT7 vector as prey. Primers are listed in Table S2.

The desired pairs of prey and bait constructs were co-transformed into Y2H Gold yeast (Takara, San Jose, CA) and plated on the SD-Leu-Trp (DDO) medium, following Takara’s instructions. Yeast clones with paired constructs that grew on the DDO were resuspended in 100 µL of sterile water, then 10 µL droplets of the culture dilutions were transferred onto DDO and SD-Leu-Trp-His (TDO) plates.

### 4.4. Bi-Molecular Fluorescence Complementation (BiFC)

The CLIBASIA_00460 coding region for the mature protein and the full-length CDS of the citrus RAD23b, c, and d genes were amplified. PCR was performed using Phusion High-Fidelity DNA Polymerase and the gene-specific primers are described in Table S2. The purified amplicons were TOPO cloned into pENTR and pDONR-201 vectors, according to the manufacturer’s directions (Thermo Fisher Scientific, Waltham, MA, USA), and verified by sequencing. The purified pDONR-201 vectors containing citrus RAD23b, c, and d were used to subclone into pEarleyGate-201-YN vector, and pENTR vector containing CLIBASIA_00460 was used to subclone into pEarleyGate202-YC vector using LR Clonase II. The full-length YFP function is complemented when targeted proteins physically interact with each other, as described by Lu et al. [46]. The resulting constructs were transformed into *Agrobacterium tumefaciens* strain LBA4404 by electroporation.

Transformed *A. tumefaciens* were grown at 28 °C for 16–24 h in LB medium containing 10 mM morpholineethanesulfonic acid (MES) pH 5.6 and 20 μM acetosyringone (AS). The cultures were pelleted and resuspended in a freshly prepared infiltration buffer (10 mM MgCl2, 10 mM MES, and 200 μM AS) to a final OD600 = 1.2. Cells were incubated in the dark for at least 4 h at room temperature. *Agrobacterium* carrying BiFC constructs (co-infiltration at v/v ratio = 1:1) were infiltrated into the intercellular spaces of leaves of 4-week-old *N. benthamiana* plants using a needleless syringe. The plants were kept at room temperature for 48 to 80 h. After infiltration, the leaves were examined using a fluorescent microscope (Axio Imager A1 microscope, Carl Zeiss Microscopy, White Plains, NY, USA) with a FITC (488 nm, green) filter for YFP signals.

### 4.5. Agrobacterium Tumefaciens-Mediated Transient Expression

The CLIBASIA_00460 coding region for the mature protein was cloned into the pEG101 plasmid and transformed into *A. tumefaciens,* as described in Levy et al. [27]. For the transient expression experiments, bacteria were grown in 3 mL of LB supplemented with kanamycin (50 µg/mL), 10 mM morpholineethanesulfonic acid (MES) and 20 µM acetosyringone for 24 h at 28 °C and 250 rpm. The cells were pelleted and resuspended in an infiltration buffer: 10 mM MES, 10 mM MgCl2, and 200 µM acetosyringone. The cells were resuspended at an optical density at 600 nm of 0.7 for those carrying CLIBASIA_00460 or AvrPto, and 0.25 for those carrying Prf^D1416V^. Agroinfiltrations were performed using disposable syringes into 5-week-old *N. benthamiana* leaves. After infiltration, the plants were maintained on light shelves at room temperature (21–24 °C). The HR was scored on a scale of 0 (no reaction) to 1 (HR reaction) for each infiltration. The co-infiltration of AvrPto and Prf^D1416V^ was used as a negative control. The experiment was repeated twice, each time infiltrations were performed in 3 leaves per plant and there were three plants per experiment.

## Data Availability

Not applicable.

## Supplementary Materials

The following supporting information can be downloaded at: https://www.mdpi.com/article/10.3390/ijms23147846/s1.
