# Supplementary material for: CLIBASIA_00460 Disrupts Hypersensitive Response and Interacts with Citrus Rad23 Proteins
Source: Int J Mol Sci. 2022 Jul 16;23(14):7846. doi: 10.3390/ijms23147846 (PMC9324546; doi:10.3390/ijms23147846)
Supplement: Supplementary file 1 [file ijms-23-07846-s001.zip › ijms-1822650-supplementary.pdf]

Table S1: DeepLoc predictions for HPE1, CLIBASIA_00460 and tomato and citrus RAD23 proteins. Only values above the probability threshold are reported.

Protein_ID	Localizations	Signals	Cytoplasm	Nucleus
HPE1 mature protein	Cytoplasm, Nucleus	Nuclear export signal	0.6373	0.6318
CLIBASIA_00460 mature protein	Cytoplasm, Nucleus	Nuclear export signal	0.6750	0.6921
SlRad23a	Cytoplasm, Nucleus	Nuclear export signal	0.7088	0.638
SlRad23c	Cytoplasm, Nucleus	Nuclear export signal	0.6745	0.5596
SlRad23d	Cytoplasm, Nucleus	Nuclear export signal	0.6697	0.5706
SlRad23e	Cytoplasm, Nucleus	Nuclear export signal	0.6907	0.5708
CsRAD23b	Cytoplasm, Nucleus	Nuclear export signal	0.7039	0.6247
CsRAD23c	Cytoplasm, Nucleus	Nuclear export signal	0.6531	0.5899
CsRAD23d	Cytoplasm, Nucleus	Nuclear export signal	0.7146	0.5884

Table S2. Primers listed in this study

Primer	Sequence 5'-3'	Purpose
CLas460-ENTR-F	CACCATGCAAGTTTATCATATCCATTC	CLas460 pENTR cloning
CLas460-ENTR-R	TTTTTTATCTTCTTCAAATAAATATTTG	CLas460 pENTR cloning
CLas460-BD-F	GCGGAATTCCAAGTTTATCATATCCATTCG	CLas460 Y2H Bait cloning
CLas460-BD-R	GCGGGATCCCTATTTTTTATCTTCTTCAA	CLas460 Y2H Bait cloning
CcRad23b-AD-F	CGCCATATGATGAAGCTCACCGTTAAGACTCT	CcRad23b Y2H prey cloning
CcRad23b-AD-R	CGCGGATCCTCAATCCTCGAAATCTCCAGCATTT	CcRad23b Y2H prey cloning
CcRad23c-AD-F	CGCGAATTCATGAAGGTTTTCGTTAAGACTTTG	CcRad23c Y2H prey cloning
CcRad23c-AD-R	CGCGGATCCTCAATCCTCAAACTCATGCATGTGA	CcRad23c Y2H prey cloning
CcRad23d-AD-F	CGCGAATTCATGAAGATTTTCGTTAAAACTCTC	CcRad23d Y2H prey cloning
CcRad23d-AD-R	CGCGGATCCTCAATCCTCAAACTCATGCATATGG	CcRad23d Y2H prey cloning
CcRad23b-BP-F	GCAGGCTTCATGAAGCTCACCGTTAAGAC	CcRad23b BiFC cloning
CcRad23b-BP-R	AGCTGGGTCATCCTCGAAATCTCCAGCATTTTC	CcRad23b BiFC cloning
CcRad23c-BP-F	GCAGGCTTCATGAAGGTTTTCGTTAAGACTTTG	CcRad23c BiFC cloning
CcRad23c-BP-R	AGCTGGGTCATCCTCAAACTCATGCATGTGA	CcRad23c BiFC cloning
CcRad23d-BP-F	GCAGGCTTCATGAAGATTTTCGTTAAAACTCTC	CcRad23c BiFC cloning
CcRad23d-BP-R	AGCTGGGTCATCCTCAAACTCATGCATATGG	CcRad23c BiFC cloning
attB-F	GGGGACAAGTTTGTACAAAAAAGCAGGCTTCATG	Attacth adapter to BP amplicon
attB-R	GGGGACCACTTTGTACAAGAAAGCTGGGTC	Attacth adapter to BP amplicon

```
SlRad23a      -MKLTVKTLKGSHFEIRVQPSDTIMAVKKNIEDVQGKDNYPCGQQLLIHNGKVLKDESTL      59
CsRAD23b      -MKLTVKTLKGSHFEIRVQPSDTVMAVKKNIEDVQGKDNYPCGQQLLIHNGKVLKDETTL      59
CsRAD23d      MMKIFVKTLKGTSFDVEVKPEDTVFDVKMKIETVQGSDVYPAAQQMLIYQGKVLKDDTTL      60
SlRad23c      -MKIFVKTLKGSHFEIEVKPEDTVADVKKNIETVQGSDVYPAAQQMLIHQGKVLKDGTTL      59
CsRAD23c      -MKVFVKTLKGTHFEIEVKPEDKVSDVKKNIETVQGSDVYPASQQMLIHQGKVLKDVTTL      59
SlRad23d      -MKIFVKTLKGTHFEIEVKPEDTVADVKKSIETVQGQDVYPAAQQMLIHQGKVLKDPTTL      59
SlRad23e      -MKIFVKTLKGTHFEIEVKPEDSVADVKKNIESVQGQDVYPAAQQMLIHQGKVLKDTTTL      59
               **: ******: *::.*:*.*.:  ** .** ***.* **..**:**::****** :**

SlRad23a      LENNVSEDGFLVVMLSKSKTASSSGTTSAQQPA-------TAANPTTTP-----------      101
CsRAD23b      ADNKVSEDGFLVVMLSKSKTLGSAGASSAQPAH--------TTPPTTAPSSNSTPPQE--      109
CsRAD23d      EENKVAENSFVVIMLTKNKRPTGEGSTTSTAPTKAPQ--SSAPTPAVTPATAPQTAVPI-      117
SlRad23c      EENKVAESNFIVIMLTKSKSSAGEGSATSTAATAK-TPQSSTAAPSSTPASTA--AQVPV      116
CsRAD23c      EENKVAENSFVVVMLTKSKVSSSGASTVSAAPANQAQTTSSAPPTSTQPTTTSQTPAPTV      119
SlRad23d      EENKVAENSFVVIMLSKNKVSTSGTSATQPAPSNSAQPATSTGQPTQ--TVAPQATAASV      117
SlRad23e      EENKVAENSFVVIMLSKNKVSSTGTSSISAALSNTAQPDGSTDQARQ--TITTPQATAAL      117
               :*:*:*..*:*:**:*.*      ::              :

SlRad23a      ----EVIPPSQ--------------APKD--VVSASDAAAASLPADDYSQAASNLVAGNN      141
CsRAD23b      ---ASVPPPAP--------------TPSIPASNVTSNVTAANANSDTYGQAASNLVAGND      152
CsRAD23d      ---ST----------------EPTPAPTPAPASASVSSVSATSESGVYGHAASNLVAGNN      158
SlRad23c      ASPAAAPSPAPAPAAAPAPALAPAPASVPVPAPALAPASAVVSGSDVYGQAASSLVAGNN      176
CsRAD23c      APPQSVPESAPT---------PAAPA--PAPAPAPA---PVSSVSDVYGQAASNLVAGSN      165
SlRad23d      APAQSAPAPAP----------APAPA--AAPAPAPA---AANPLTDVYGQAASNLVAGST      162
SlRad23e      --------PQS----------ASESA--PTPAPVPA---AASSVTDVYDQAASNLVAGSN      154
                                       :              .   :. *.:***.****.

SlRad23a      LEQTIQQLMDMGGGSWDKETVTRALRAAYNNPERAVDYLYSGIPETAEVSVPVARGGVNS      201
CsRAD23b      LEQTIQQIMDMGGGTWDKETVTRALQAAYNNPERAVDYLYSGIPETAEVAVPVAHFPASQ      212
CsRAD23d      LEGAIQQILDMGGGTWDRDTVVRALRAAYNNPERAVEYLYSGIPEQAEVQ-PVARAPG--      215
SlRad23c      LDGAIQQILDMGGGTWDRETVVRALRAAFNNPERAVEYLYSGIPEAAEVP-PVGGSPP--      233
CsRAD23c      LEATVQQILDMGGGSWDRETVIRALRAAYNNPERAVEYLYSGIPEQTAVP-PVARASAG-      223
SlRad23d      LETTVQQILDMGGGSWERDTVVRALRAAYNNPERAIEYLYSGIPEQAEIP-PVAPA----      217
SlRad23e      LETTVQQILDMGGGSWDRDTVVRALRAAYNNPERAVDYLYSGIPEQTEIP-PVARAPAAP      213
              *: ::**::*****:*:::** ***:**:******::******** : :  **.

SlRad23a      ---------AAV------PTAAPIAPSSGAPNSAPLNLFPQENVA-GGGGAGLGSLDFLR      245
CsRAD23b      ---------AAE------TGAAGAAPVSGVPNSSPLNMFPQETLS-GAPASGLGSLDFLR      256
CsRAD23d      ------NGQAANSPTQMPQPTQPAPVTSSGPNANPLDLFPQGLPDVGSGAAGAGSLDFLR      269
SlRad23c      ------SVQAGNQPAQPQPAAQPAPVPAGGPNADPLNLFPQGLPSVGSNTAGANTLDFLR      287
CsRAD23c      -------GQAGNPPAQTQAQQPAAPAPTSGPNANPLDLFPQGLPNMGSN-AGAGTLDFLR      275
SlRad23d      ------SGQAVNPP-------VQASQPSSGPNANPLDLFPQGLPNVGAN-AGAGNLDFLS      263
SlRad23e      AVTAPASAQAINPAAQDA---SQLAVPSSGPNANPLDLFPQGLTNAGSN-AGAGNLDFLR      269
                       *                 :. **: **::***     *.  :* ..****

SlRad23a      NNQQFQALRSMVQANPQILQPMLQELGKQNPQLLRSIQEHDQEFLQLINEPVDGSDGD-M      304
CsRAD23b      NNQQFQALRSMVQSNPQILQPMLQELGKQNPQLLRLIQEHQAEFLQLINEPVDGSEGD-M      315
CsRAD23d      NSPQFQVLRAMVQANPQLLQPMLQELGKQNPQLVRLIQEHQADFLRLINEPVQGGEGN-L      328
SlRad23c      NSQQFQALRAMVQANPQILQPMLQELGKQNPHLMRLIQEHQADFLRLINEPVEGGEGNNV      347
CsRAD23c      NSQQFQALRTMVQANPQILQPMLQELGKQNPHLMRLIQEHQTDFLRLINEPVEGGEGN-V      334
SlRad23d      NSPQFQALRAMVQANPQILQPMLQELGKQNPQLMRLIQEHQADFLRLINEPVEG-EGN-V      321
SlRad23e      NSPQFQALRAMVQANPQILQPMLQELGKQNPHLMRLIQEHQPDFLRLINEPVEG-EGN-V      327
              *. ***.**:***:***:*************:*:* ****: :**:******:* :*: :

SlRad23a      FDQAEQEIPHTVSVTPEEQEVIERLEAMGFDRALVIEAFLACDRNEELAANYLLEHAGDY      364
CsRAD23b      FDQPEQDMPHAINVTPAEQEAIQRLEAMGFDRALVIEAFLACDRNEELAANYLLENAGDF      375
CsRAD23d      GDQLAGLMPQSIQVTPEEREAIERLEAMGFDRAIVLEVFFACNKNEELAANYLLDHMHEF      388
SlRad23c      LGQLAAAMPQAISVTPEEREAIERLEQMGFDHDLVLEVFFACNKNEELAANYLLDHIHEF      407
CsRAD23c      LGQLASAMPQAVTVTPEEREAIERLEAMGFDRALVLEVFFACNKNEELAANYLLDHMHEF      394
SlRad23d      PGQPAGAIPQAVTVTPEEREAIERLEAMGFDRALVLQVYFACNKNEEMAANYLLDHMHEF      381
SlRad23e      LGQTAGAIPQAVTVTPEEREAIERLEAMGFDRALVLEVYFACNKNEELAANYLLDHLHEF      387
               .*    :*::: *** *:*.*:*** ****: :*::.::**::***:******::   ::

SlRad23a      ED 366
CsRAD23b      ED 377
CsRAD23d      ED 390
SlRad23c      EE 409
CsRAD23c      ED 396
SlRad23d      DE 383
SlRad23e      DE 389
              ::
```

Figure S1: Alignment of *S. lycopersicum* and *C. sinensis* RAD23 proteins. Amino acid residues are colored according to their physiochemical properties: red = small (small+ hydrophobic including aromatic -Y), blue = acidic, magenta = basic -H , green = hydroxyl + sulfhydryl + amine + G. Consensus symbols are as follows: ‘*’ (asterisk) indicates positions which have a single, fully conserved residue; ‘:’ (colon) indicates conservation between groups of strongly similar properties, ‘.’ (period) indicates conservation between groups of weakly similar properties.

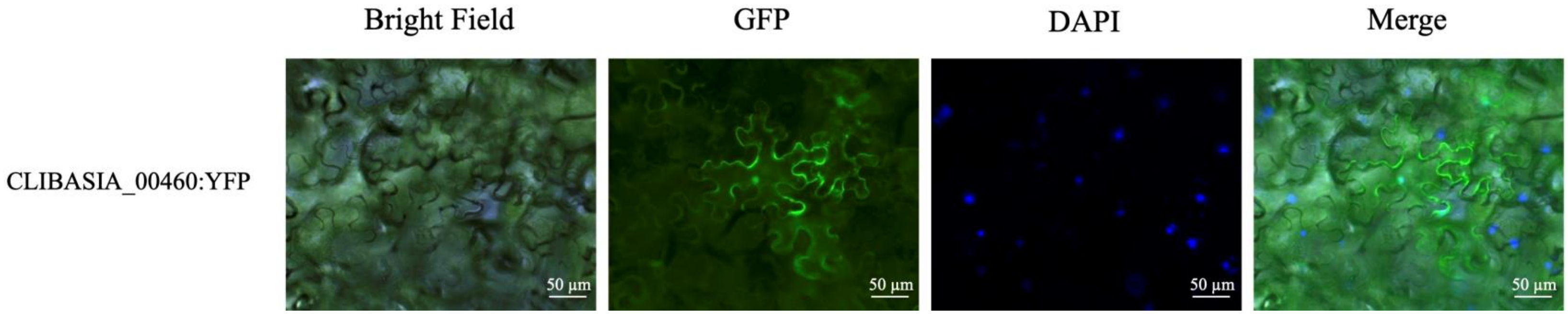

Figure S2. The CLIBASIA00460 mature protein lacking the N-terminal signal peptide was cloned with an N-terminal fusion to GFP and visualized by fluorescence microscopy 72 h after transient expression in *N. benthamiana*. pEG101:CLIBASIA00460 localized at the nucleus and cytosol of epidermal cells. Scale bar = 50 µm.
